# Early decompressive hemicraniectomy in thrombolyzed acute ischemic stroke patients from the international ENCHANTED trial

**DOI:** 10.1038/s41598-021-96087-z

**Published:** 2021-08-13

**Authors:** Chao Xia, Xia Wang, Richard I. Lindley, Candice Delcourt, Xiaoying Chen, Zien Zhou, Rui Guo, Cheryl Carcel, Alejandra Malavera, Zeljka Calic, Grant Mair, Joanna M. Wardlaw, Thompson G. Robinson, Craig S. Anderson

**Affiliations:** 1 Huaxi MR Research Center (HMRRC), Department of Radiology, West China Hospital, Sichuan University, Chengdu, Sichuan People’s Republic of China; 2 Department of Neurosurgery, West China Hospital, Sichuan University, Chengdu, Sichuan People’s Republic of China; 3 The George Institute for Global Health, Faculty of Medicine, University of New South Wales, Sydney, NSW Australia; 4 Westmead Applied Research Centre, University of Sydney, Sydney, NSW Australia; 5 The George Institute for Global Health, Missenden Rd., PO Box M201, Camperdown, NSW 2050 Australia; 6 Department of Clinical Medicine, Faculty of Medicine, Health and Human Sciences, Macquarie University, Macquarie Park, NSW Australia; 7 Department of Radiology, Ren Ji Hospital, School of Medicine, Shanghai Jiao Tong University, Shanghai, People’s Republic of China; 8 Department of Neurology, Royal Prince Alfred Hospital, Sydney Health Partners, Sydney, NSW Australia; 9 Division of Neuroimaging Sciences, Edinburgh Imaging and Centre for Clinical Brain Sciences, University of Edinburgh, Edinburgh, UK; 10 Department of Cardiovascular Sciences and NIHR Leicester Biomedical Research Centre, University of Leicester, Leicester, UK; 11 The George Institute China at Peking University Health Science Centre, Beijing, People’s Republic of China; 12 Heart Health Research Center, Beijing, People’s Republic of China

**Keywords:** Neurology, Neurological disorders

## Abstract

Decompressive hemicraniectomy (DHC) can improve outcomes for patients with severe forms of acute ischemic stroke (AIS), but the evidence is mainly derived from non-thrombolyzed patients. We aimed to determine the characteristics and outcomes of early DHC in thrombolyzed AIS participants of the international Enhanced Control of Hypertension and Thrombolysis Stroke Study (ENCHANTED). Post-hoc analyses of ENCHANTED, an international, partial-factorial, open, blinded outcome-assessed, controlled trial in 4557 thrombolysis-eligible AIS patients randomized to low- versus standard-dose intravenous alteplase (Arm A, n = 2350), intensive versus guideline-recommended blood pressure control (Arm B, n = 1280), or both (Arms A + B, n = 947). Logistic regression models were used to identify baseline variables associated with DHC, with inverse probability of treatment weights employed to eliminate baseline imbalances between those with and without DHC. Logistic regression was also used to determine associations of DHC and clinical outcomes of death/disability, major disability, and death (defined by scores 2–6, 3–5, and 6, respectively, on the modified Rankin scale) at 90 days post-randomization. There were 95 (2.1%) thrombolyzed AIS patients who underwent DHC, who were significantly younger, of non-Asian ethnicity, and more likely to have had prior lipid-lowering treatment and severe neurological impairment from large vessel occlusion than other patients. DHC patients were more likely to receive other management interventions and have poor functional outcomes than non-DHC patients, with no relation to different doses of intravenous alteplase. Compared to other thrombolyzed AIS patients, those who received DHC had a poor prognosis from more severe disease despite intensive in-hospital management.

## Introduction

Stroke is a leading cause of premature loss of productive life, estimated to have caused several million deaths worldwide in 2017, half due to acute ischemic stroke (AIS)^1^. Reperfusion therapy with intravenous thrombolysis (i.e. recombinant tissue plasminogen activator [rt-PA] or alteplase) and more recently endovascular clot retrieval (EVT) are now standard treatments that can improve the chances of functional recovery when administered within several hours of symptom onset^2^. However, thrombolysis has more limited utility in AIS due to large vessel occlusion, EVT is not widely available, and the benefits of both are offset by increased risks of intracranial hemorrhage (ICH)^3–5^. In patients with malignant hemispheric AIS, particularly those presenting late or unable to access mechanical thrombectomy, decompressive hemicraniectomy (DHC) has also been shown to improve outcomes from reducing intracranial pressure and preventing brain herniation^6^. However, the evidence for the benefits of DHC has mainly been derived from trials of young to middle-aged non-thrombolyzed adults with cerebral edema and mass effect from hemispheric AIS^7–11^, and there is limited data on outcomes from early DHC after intravenous thrombolysis in a broader range of patients where the risk of ICH from the treatment and surgery are high. Moreover, as access to, and criteria for, the use of DHC in AIS varies widely within and between countries, there is uncertainty over its benefit in ‘real world’ clinical practice^12,13^. The aim of this study was to characterize the use of DHC and its relation to clinical outcomes in thrombolyzed AIS patients who participated in the international Enhanced Control of Hypertension and Thrombolysis Stroke Study (ENCHANTED).

## Methods

### Study design

ENCHANTED was an international, multicenter, prospective, partial-factorial, randomized, open, blinded-endpoint trial that investigated the effectiveness of low-dose (0.6 mg/kg) versus standard-dose (0.9 mg/kg) intravenous alteplase and intensive (systolic target < 140 mmHg) versus standard (< 180 mmHg) blood pressure (BP) lowering in 4557 thrombolysis-eligible and treated AIS patients between March 2012 and April 2018^14–18^. Patients were recruited at 111 clinical centers in five Asian countries (including China, Korea, Vietnam) and seven non-Asian countries (including United Kingdom, Brazil, Chile). Patients could participate in one or both arms of the study, according to inclusion/exclusion criteria, on a background of management according to best practice guidelines. The study was approved by the ethics committees of participating centers (see Supplementary list)^16^, and all patients (or legally appropriate surrogates) provided written informed consent. All methods were performed in accordance with the relevant guidelines and regulations. The study is registered at Clinicaltrials.gov (NCT01422616).

### Procedures

Demographic, medical history, and clinical characteristic data, including the severity of neurological impairment on the National Institute of Health Stroke Scale (NIHSS), were obtained at the time of enrollment (baseline). Imaging data on computerized tomography (CT), magnetic resonance imaging (MRI), and angiography were collected in DICOM format, anonymized, and centrally analyzed by at least two independent expert assessors blind to clinical information and treatment assignments. DHC was defined as hemicraniectomy or decompressive surgery recorded on the study case record form at 7 days (or hospital discharge, if sooner) or as reported according to standard criteria for a serious adverse event (SAE) to the end of the 90-day follow-up. In the case of multiple SAEs, only SAEs related to surgical decompression were included in the analyses. All patients not known to have died were followed up in-person or by telephone by trained independent researchers at 90 days post-randomization. The primary outcome for these analyses was death or any disability, defined by scores 2–6 on the modified Rankin scale (mRS). Other outcomes included death/major disability and death (mRS scores 3–6 and 6, respectively).

### Statistical analysis

Predictors of DHC were assessed in multivariable logistic regression models that included age, sex, Asian ethnicity, and all significant variables (P < 0.05) in univariable analysis. Considering disequilibrium and unbalanced variables between two groups, a propensity score analysis was also undertaken whereby a propensity score for each subject was obtained in a logistic regression model that included age, sex, ethnicity (Asian vs. non-Asian), hypertension, atrial fibrillation, antithrombotic therapy, lipid-lowering therapy, baseline NIHSS score, and a final diagnosis of large artery occlusion due to significant atheroma. The inverse probability of treatment weighting (IPTW) was used as the primary strategy to balance the baseline data^19^, which was examined using an absolute standardized mean difference within an acceptable margin of 0.2^20^, indicating well-balanced distributions of covariates (see Supplementary Fig. S1 online). Stabilized weights were then incorporated into logistic regression models to determine associations of surgical intervention and outcomes. Data are reported with odds ratio (OR) and 95% confidence interval (CI). A 2-sided P value < 0.05 was set as a standard level of significance. All analyses were performed using SAS 9.3 software (SAS Institute, North Carolina, US).

## Results

Of 4557 included patients (2350, 1280, 947 in Arms A, B, and A + B, respectively), 95 (2.1%) underwent DHC. Of these surgical patients, the timing of intervention for 74 (78%) was within 6 days of randomization, including 73 (99% with available data of timing) who underwent DHC within two days of neurological deterioration (see Supplementary Fig. S2 online). The indications for DHC were malignant brain edema with/without mass effect (36, 38%), and ICH (27, 28%), with 32 (34%) patients without such information. Table 1 shows that compared to other patients, those who underwent DHC were more likely to be of non-Asian ethnicity, have a history of hypertension, hypercholesterolemia, and prior use of lipid-lowering therapy, higher baseline NIHSS score, and a final diagnosis of proximal cerebral vessel occlusion identified on CT/MRI angiography. After applying IPTW with a stabilized weight method, only three baseline categorical covariates (atrial fibrillation, prior antithrombotic therapy, and lipid-lowering therapy) were not well balanced between the two groups (all P < 0.05) (Table 1).

**Table 1:** Baseline characteristics in relation to decompressive hemicraniectomy for acute ischemic stroke, according to the use of inverse probability of treatment weighting.

	Unweighted populations		P value*	Weighted populations		P value*
	Decompressive hemicraniectomy			Decompressive hemicraniectomy		
	Yes (n = 95)	No (n = 4462)		Yes (n = 87)	No (n = 4349)	
Time from stroke onset to randomization, h	2.8 (2.2–3.9)	2.9 (2.1–3.7)	0.75	3.5 (2.4–4.3)	2.9 (2.1–3.7)	−^†^
Age, year	65 (11)	66 (13)	0.23	68 (11)	66 (13)	−^†^
Female	31 (32.6)	1691 (37.9)	0.30	30 (34.5)	1640 (37.7)	0.57
Asian ethnicity	51 (53.7)	2968 (66.5)	0.01	53 (60.9)	2894 (66.5)	0.27
Hypertension	71/95 (74.7)	2866/4451 (64.4)	0.04	62 (71.3)	2813 (64.7)	0.20
Previous stroke	12 (12.6)	811 (18.2)	0.17	9 (10.3)	793 (18.2)	0.08
Atrial fibrillation	23/95 (24.2)	785/4447 (17.7)	0.10	22 (25.3)	763 (17.5)	0.04
Coronary artery or other heart diseases	25/95 (26.3)	886/4451 (19.9)	0.12	21 (24.1)	855 (19.7)	0.29
Diabetes mellitus	21/95 (22.1)	907/4451 (20.4)	0.68	12 (13.8)	889 (20.4)	0.15
Hypercholesterolemia	23/95 (24.2)	684/4451 (15.4)	0.02	17 (19.5)	675 (15.5)	0.31
Current smoker	25/95 (26.3)	983/4445 (22.1)	0.33	16/87 (18.4)	957/4345 (22.0)	0.36
Pre-stroke absence of symptoms	78/94 (83.0)	3700/4449 (83.2)	0.96	73/85 (85.9)	3633/4348 (83.6)	0.60
Antihypertensive therapy	52 (54.7)	2092 (46.9)	0.13	47 (54.0)	2042 (47.0)	0.21
Antithrombotic therapy	30/94 (31.9)	1043/4449 (23.4)	0.06	31 (35.6)	1024 (23.5)	0.01
Lipid-lowering therapy	29/94 (30.9)	817/4448 (18.4)	< 0.01	25 (28.7)	811 (18.6)	0.02
Systolic BP, mmHg	153 (18)	153 (19)	0.68	156 (16)	153 (19)	–^†^
NIHSS score	13 (6–17)	8 (5–13)	< 0.01	8 (5–14)	8 (5–13)	–^†^
NIHSS score ≥ 15	38 (40.0)	889 (19.9)	< 0.01	21 (24.1)	865 (19.9)	0.38
Cerebral infarction with mass effect	3/95 (3.2)	55/4451 (1.2)	0.12	2 (2.3)	53 (1.2)	0.63
Proximal clot on CT/MRI angiogram	29/94 (30.9)	597/4409 (13.5)	< 0.01	13 (14.9)	603 (13.9)	0.73
**Final diagnosis**						
Large artery occlusion from atheroma	53/94 (56.4)	1751/4400 (39.8)	< 0.01	31 (35.6)	1748 (40.2)	0.21
Small vessel lacunar disease	4/94 (4.3)	1053/4400 (23.9)		15 (17.28)	1026 (23.6)	
Cardioembolism	18/94 (19.1)	782/4400 (17.8)		18 (20.7)	768 (17.7)	
Other	19/94 (20.2)	814/4400 (18.5)		22 (25.3)	807 (18.6)	
Randomized low-dose alteplase	32/66 (48.5)	1618/3220 (50.2)	0.78	20/47 (42.6)	1568/3128 (50.1)	0.55
Randomized intensive BP lowering	18/42 (42.9)	1063/2154 (49.4)	0.41	24/51 (47.1)	1045/2112 (49.5)	0.81

Data are n (%), mean (SD), or median (IQR).

*BP* blood pressure, *CT* computed tomography, *MRI* magnetic resonance imaging, *NIHSS* National Institute of Health Stroke Scale.

*P values based on χ^2^ test, Fisher’s exact test, or Wilcoxon test.

^†^Not applicable for continuous variables in weighted populations.

Table 2 shows the independent predictors for DHC were younger age (OR 0.98, 95% CI 0.96–0.99; P < 0.01), non-Asian ethnicity (OR 0.49, 95% CI 0.30–0.79; P < 0.01), lipid-lowering therapy (OR 2.11, 95% CI 1.29–3.46; P < 0.01), severe neurological impairment (OR 1.08, 95% CI 1.05–1.10; P < 0.01), and large artery occlusive AIS (OR 2.37, 95% CI 1.52–3.70; P < 0.01).

**Table 2:** Independent predictors associated with decompressive hemicraniectomy.

	Decompressive hemicraniectomy			
	OR (95% CI)	P value	aOR (95% CI)*	P value
Time from onset to randomization, h	1.04 (0.86–1.26)	0.66		
Age, year	0.99 (0.98–1.01)	0.31	0.98 (0.96–0.99)	< 0.01
Female	0.79 (0.52–1.22)	0.30		
Asian ethnicity	0.58 (0.39–0.88)	0.01	0.49 (0.30–0.79)	< 0.01
Hypertension	1.64 (1.03–2.61)	0.04		
Previous stroke	0.65 (0.35–1.20)	0.17		
Atrial fibrillation	1.49 (0.93–2.40)	0.10		
Coronary artery and other heart diseases	1.44 (0.91–2.28)	0.12		
Diabetes mellitus	1.11 (0.68–1.81)	0.68		
Hypercholesterolemia	1.76 (1.09–2.83)	0.02		
Current smoker	1.26 (0.79–2.00)	0.33		
Prestroke absence of symptoms	1.01 (0.59–1.75)	0.96		
Antihypertensive therapy	1.37 (0.91–2.06)	0.13		
Antithrombotic therapy	1.53 (0.99–2.38)	0.06		
Lipid-lowering therapy	1.98 (1.27–3.09)	< 0.01	2.11 (1.29–3.46)	< 0.01
Systolic BP, mmHg	1.00 (0.99–1.01)	0.77		
NIHSS score	1.07 (1.04–1.10)	< 0.01	1.08 (1.05–1.10)	< 0.01
Cerebral infarction with mass effect	2.61 (0.80–8.48)	0.11		
Large artery occlusion due to significant atheroma	1.96 (1.30–2.95)	< 0.01	2.37 (1.52–3.70)	< 0.01

*aOR* adjusted odds ratio, *BP* blood pressure, *CI* confidence interval, *NIHSS* National Institute of Health Stroke Scale, *OR* odds ratio.

*Included all significant variables in univariable analysis, and age, sex, and Asian ethnicity.

During the first seven days of hospital admission, patients who underwent DHC had more cerebral angiograms, intravenous BP lowering treatment, admission to monitored and organized care areas (i.e., intensive care unit), and other interventions (intubation and ventilation, treatment of pyrexia, nasogastric feeding, and use of compression stockings for venous thromboembolism prophylaxis), but had less use of traditional intravenous Chinese medicine (Table 3).

**Table 3:** Other management in the first 7 days of hospital admission.

	Decompressive hemicraniectomy		P value*
	Yes (n = 95)	No (n = 4462)	
Cerebral angiogram	13/95 (13.7)	243/4451 (5.5)	< 0.01
Intra-arterial alteplase	2/13 (15.4)	34/242 (14.1)	0.89
Any IV BP lowering in first 24 h	20/40 (50.0)	668/1381 (48.3)	0.83
Any IV BP lowering in days 2–7	37/93 (39.8)	924/4380 (21.1)	< 0.01
Systolic BP at 24 h	140 (21)	138 (19)	0.01
Intubation and ventilation	48/92 (52.2)	179/4381 (4.1)	< 0.01
Fever occurrence	47/92 (51.1)	765/4380 (17.5)	< 0.01
Fever treated	43/88 (48.9)	646/3964 (16.3)	< 0.01
Nasogastric feeding	54/92 (58.7)	737/4380 (16.8)	< 0.01
Mobilization by physiotherapist	47/92 (51.1)	1926/4380 (44.0)	0.17
Compression stockings	21/92 (22.8)	363/4379 (8.3)	< 0.01
Subcutaneous heparin	20/95 (21.1)	841/4462 (18.9)	0.59
Any antithrombotic agent in first 24 h	17/95 (17.9)	730/4445 (16.4)	0.70
Traditional IV Chinese medicine	11/92 (12.0)	1421/4380 (32.4)	< 0.01
IV corticosteroids	3/92 (3.3)	89/4380 (2.0)	0.44
Acute stroke unit admission	58/93 (62.4)	2432/4380 (55.5)	0.19
ICU admission	58/93 (62.4)	943/4379 (21.5)	< 0.01
Any rehabilitation given	40/92 (43.5)	2183/4381 (49.8)	0.23

Data are n (%) or mean (SD).

BP blood pressure, ICU intensive care unit, IV intravenous.

*P values are based on χ^2^ test, Fisher’s Exact test, or Wilcoxon test.

**Figure 1:**
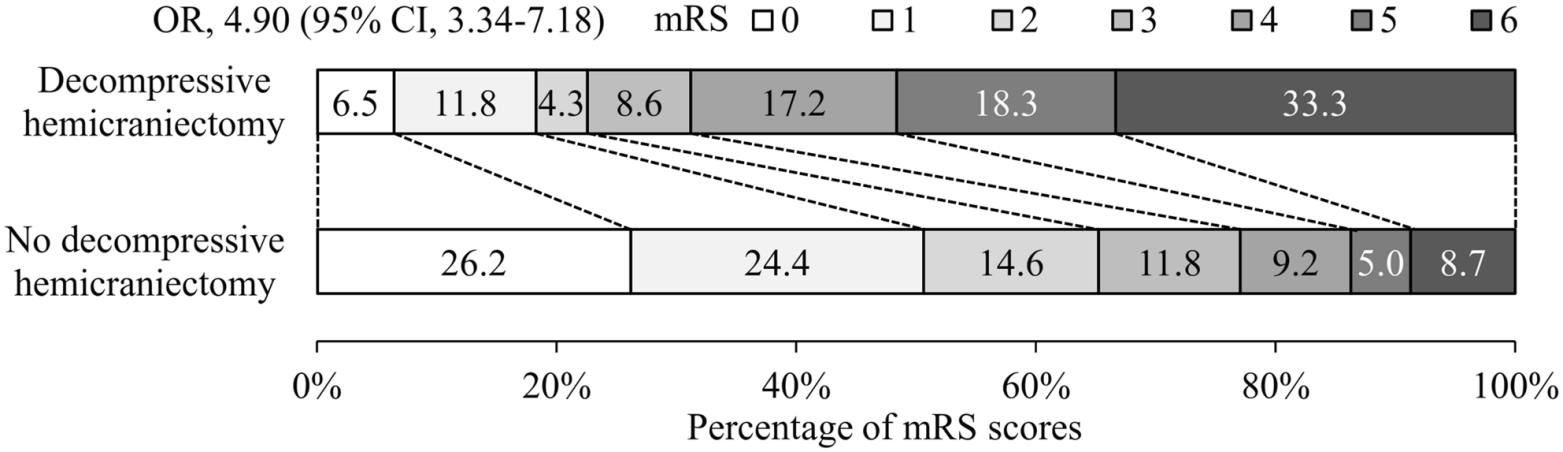
Functional outcomes on the modified Rankin scale at 90 days by groups. The figure shows the distribution of scores on the modified Rankin scale at 90 days with scores ranging from 0 to 6. *CI* confidence interval, *OR* odds ratio, *mRS* modified Rankin scale.

Overall, there were 2231 (49.0%) patients who were either dead or disabled at 90 days. When compared to other patients, those who received DHC had worse patterns of recovery with 82% death or dependency (Table 4; Fig. 1). There were no differences in clinical outcomes between patients with low-dose versus standard-dose intravenous alteplase treatment prior to DHC (see Supplementary Table S1 online). Amongst all 4557 in the trial, 81 (2%) patients had ICH based on SITS-MOST criteria, and this had occurred before DHC in these patients (27, 28%).

**Table 4:** Decompressive hemicraniectomy and clinical outcomes at 90 days.

	Decompressive hemicraniectomy		Multivariate regression		Propensity score	
	Yes (n = 93)	No (n = 4365)	OR (95% CI)	P value	OR (95% CI)	P value
Death/disability (mRS 2–6)*	76 (81.7)	2155 (49.4)	3.53 (1.99–6.27)	< 0.01	3.86 (2.29–6.51)	< 0.01
Death/major disability (mRS 3–6)*	72 (77.4)	1518 (34.8)	5.74 (3.35–9.82)	< 0.01	4.40 (2.76–7.02)	< 0.01
Death (mRS 6)*	31 (33.3)	381 (8.7)	5.43 (3.28–8.97)	< 0.01	4.95 (3.07–7.98)	< 0.01
Shift in mRS scores*			4.90 (3.34–7.18)	< 0.01	4.89 (3.34–7.16)	< 0.01
0	6 (6.5)	1144 (26.2)				
1	11 (11.8)	1066 (24.4)				
2	4 (4.3)	637 (14.6)				
3	8 (8.6)	516 (11.8)				
4	16 (17.2)	403 (9.2)				
5	17 (18.3)	218 (5.0)				
6	31 (33.3)	381 (8.7)				

*CI* confidence interval, *mRS* modified Rankin scale, *OR* odds ratio.

*The mRS evaluates global disability; scores range from 0 (no symptoms) to 6 (death). A score of 2 to 5 indicates some degree of disability.

## Discussion

In this study, derived from an international multicenter clinical trial database, we have shown that approximately only one in 50 thrombolyzed AIS patients receive DHC treatment. Compared to other patients, those who received DHC were significantly younger, of non-Asian ethnicity, more often on lipid-lowering therapy, had a severe neurological impairment, and an AIS due to large vessel occlusion. Despite receiving this life-saving intervention and high use of ancillary management strategies, DHC patients had a poor prognosis due to their severe form of AIS.

The pattern of surgical intervention for AIS has changed as EVT has evolved since the first randomized controlled trials were published in 2014 and 2015. Nationwide US trends suggest EVT has increased from 3.4 to 9.8% from 2012 to 2016, whereas DHC for those with malignant cerebral edema has declined from 11.4 to 4.8%^21^. A retrospective study in Germany has also observed a near 50% reduction, from 17.4 to 8.2%, in DHC after the introduction of stent retrievers for AIS^22^. The low frequency (2.1%) of DHC in our study likely reflects the selection of thrombolyzed AIS patients for a clinical trial, but the true rate may be even lower following the introduction of EVT in routine clinical practice.

As outlined in the most recent American Heart Association/American Stroke Association (AHA/ASA) guidelines, DHC is recommended for AIS patients who deteriorate neurologically from malignant brain swelling from middle cerebral artery infarction within 48 h despite medical therapy^6^. In addition to brain swelling, our study found that ICH and mass effect were the two key clinical indications of DHC. Regarding the timing of surgery, 19% of patients underwent surgery ≥ 7 days after their randomization because these patients had delayed neurological deterioration later than 7 days after randomization, which required DHC intervention. However, DHC was performed on these patients as an emergent intervention within 48 h of neurological deterioration. Thus, decisions over the use of DHC and other surgical and medical management were according to the attending clinician’s decision based on the available resources, family wishes, and clinical practice in the context of local interpretation of guideline recommendations.

Our study demonstrated that factors associated with DHC included younger age, severe neurological impairment, non-Asian ethnicity, and prior use of lipid-lowering therapy. Considering widely accepted indications for DHC in clinical practice and the literature, the former two criteria are not surprising. It is not clear why an association was found for non-Asian ethnicity and DHC. Although differing stroke pathology and mechanisms between Asians and non-Asians is a possible explanation, the association remained in analyses limited to large vessel occlusion. Other explanations for non-Asian ethnicity and prior use of lipid-lowering therapy include chance and residual confounding in relation to the limitations of these secondary subgroup analyses.

Despite the short serum half-life of rt-PA (4–6 min), its fibrinolytic effect may last up to 24–48 h^23^. As symptomatic ICH is the most severe complication of intravenous thrombolysis with rt-PA^24^, a major concern in the subsequent use of DHC is the potential for perioperative ICH. Although young to middle-aged patients appear to derive the main benefits of DHC in malignant hemispheric AIS, the randomized evidence is from non-thrombolyzed patients^7–11^. There were few studies with small numbers of patients that suggest post-thrombolysis (single 0.6 mg/kg dose, not exceeding 60 mg) DHC is safe and without an apparent excess of ICH^25–28^. Our study of older patients (mean age 66 years) suggests a poor outcome for DHC patients after thrombolysis, but the outcomes are compatible with prior DHC trials. Moreover, there was no apparent differential influence of the dose of rt-PA on outcomes from DHC, but the numbers are small and there is likely to be indication bias and residual confounding complicating our data. While the frequency of symptomatic ICH after thrombolysis ranges from 2 to 7% in prospective stroke registries^29^, the figure for hemorrhagic transformation of a large ischemic lesion is much higher in those who proceed to DHC in AIS^27,30^. Furthermore, a recent study has shown similar rates of hemorrhagic transformation in those who had DHC where they had received thrombolysis or not (64.3% versus 66.7%; P = 0.906)^25^. Yet, our results showed a big difference in ICH rates for those who underwent DHC (28.4%) versus those who did not (1.2%), demonstrating that ICH was considered as an indication for DHC by the local clinicians.

The strengths of our study are the inclusion of a broad range of patients from different health care settings globally who had systematic prospective assessments as part of a clinical trial. Another novel aspect of this study is providing evidence of the effects of DHC in the context of thrombolyzed and older patients. However, while we undertook both multivariable and propensity score adjusted analyses, the overall numbers of DHC patients were small and limited our ability to completely adjust for baseline prognostic variables. Moreover, these analyses were post-hoc, non-randomized, and derived from a clinical trial population of thrombolyzed AIS patients with predominantly mild-moderate neurological severity, and data on DHC indications were based on reporting by the investigators rather than primary data collected as part of the trial. Unfortunately, detailed information on early ischemic change and hemorrhagic transformation of the infarct was lacking, except for the Alberta Stroke Program Early CT Score (ASPECTS) for participants in Arm A (see Supplementary Fig. S2 online). These findings therefore need to be interpreted with caution.

## Conclusions

In summary, our analysis of a large and broad range of thrombolyzed AIS patients has shown that DHC is associated with a poor outcome in those who receive this intervention for severe neurological deterioration. These data may guide clinical decision-making and the counseling of patients and family members.

## Supplementary Information

Supplementary Information.

## Data Availability

The datasets generated and/or analyzed during the current study are available from the corresponding author upon reasonable request.
