# Supplementary material for: Early decompressive hemicraniectomy in thrombolyzed acute ischemic stroke patients from the international ENCHANTED trial
Source: Sci Rep. 2021 Aug 13;11:16495. doi: 10.1038/s41598-021-96087-z (PMC8363671; doi:10.1038/s41598-021-96087-z)
Supplement: Supplementary file 1 — Supplementary Information. [file 41598_2021_96087_MOESM1_ESM.pdf]

Supplementary material

Early decompressive hemicraniectomy in thrombolyzed acute ischemic stroke patients from the international ENCHANTED trial

Chao Xia[1, 2, 3], Xia Wang[3], Richard I. Lindley[4, 5], Candice Delcourt[3, 5, 6], Xiaoying Chen[3, 5], Zien Zhou[3, 7], Rui Guo[2], Cheryl Carcel[3, 5, 8], Alejandra Malavera[3], Zeljka Calic[3], Grant Mair[9], Joanna M. Wardlaw[9], Thompson G. Robinson[10], Craig S. Anderson[3, 5, 8, 11, 12, *]

***Corresponding author:** Craig S Anderson, The George Institute for Global Health, PO Box M201, Missenden Rd., Camperdown, NSW 2050, Australia; Email: canderson@georgeinstitute.org.au.

Supplementary List of the ENCHANTED participating centers.

Australia: Royal North Shore Hospital; Royal Prince Alfred Hospital; Western Hospital; John Hunter Hospital.

Brazil: Hospital de Clínicas de Porto Alegre; Hospital das Clínicas da Faculdade de Ribeirão Preto - Universidade de São Paulo; Hospital Municipal São José- Joinville – SC; Hospital das Clínicas UNESP Botucatu; Hospital de Base de São Josédo Rio Preto; Hospital São Paulo/Universidade Federal de São Paulo.

Chile: Clinica Alemana de Santiago; Clínica Alemana de Temuco; Hospital del Salvador; Hospital Barros Luco Trudeau.

China: Xuzhou Central Hospital; The Second Affiliated Hospital of Soochow University; Yutian County Hospital; No.263 Hospital of PLA; Baotou Central Hospital; Nanjing First Hospital; The Second Affiliated Hospital of Xuzhou Medical College; The First Affiliated Hospital of Wenzhou Medical University; Inner Mongolia North Heavy Industries Group Hospital; Hejian People's Hospital; The First Affiliated Hospital of Baotou Medical College; The Affiliated Hospital of Xuzhou Medical College; Yangquan Coalmine Group General Hospital; The Affiliated Jiangyin Hospital of Southeast University; West China Hospital, Sichuan University; Inner Mongolia Bao Gang Hospital; The Second Affiliated Hospital & Yuying Children's Hospital of Wenzhou Medical University; 85 Hospital of People's Liberation Army; Affiliated Hospital of Jining Medical University; Xiangya Hospital of South Central University; Zhongshan Hospital, Xiamen University; JiuJiang University Clinical Medical College / Jiujiang University hospital; Sichuan Academy of Medical Sciences & Sichuan Provincial People's Hospital; Nanjing Drum Tower Hospital the Affiliated Hospital of Nanjing University Medical School; The Second Hospital of Hebei Medical University; The Second Affiliated Hospital of Guangzhou Medical University; The Nuclear Industry 416 Hospital; Affiliated Zhongshan Hospital of Dalian University. Hong Kong: Prince of Wales Hospital. Taiwan: Chang Gung Memorial Hospital, Linkou; Mackay Memorial Hospital; Kaohsiung Chang Gung Memorial Hospital; Chi Mei Medical Center; Kaohsiung Medical University Chung-Ho Memorial Hospital; Cheng Ching Hospital; Chiayi Chang Gung Memorial Hospital; Kaohsiung Veterans General Hospital.

Colombia: Fundación Cardiovascular de Colombia; Clínica de Marly.

Italy: Ospedale di Cittàdi Castello; Ospedale di Branca; San Giovanni Battista Focigno.

Korea: Soonchunhyang University Hospital; Dong-A University Medical Center; ASAN Medical Center; Eulji Medical Center; Kyungpook National University Hospital; Inje University Busan Paik Hospital; Inha University Hospital; Seoul St's Mary's Hospital; Chungnam National University Hospital; Ewhawomans University Mokdong Hospital; Korea University Guro Hospital; Dongguk University Iisan Hospital.

Norway: Sykehuset Innlandet HF-Seksjon Gjovik.

Singapore: National University Hospital.

Thailand: King Chulalongkom Memorial Hospital.

United Kingdom: University Hospitals of the North Midlands NHS Trust; King's College Hospital; University College Hospital NHS Foundation Trust; Nottingham University Hospitals; St. George's Hospital; Charing Cross Hospital; Royal Victoria Infirmary; Princess Royal University Hospital; Salford Royal NHS Foundation Trus; Derby Teaching Hospitals NHS Foundation Trust; James Cook University Hospital; York Teaching Hospital NHS FT; Leicester Royal Infirmary; Addenbrookes Hospital; Doncaster Royal Infirmary; Northumbria Healthcare; University Hospitals Southampton NHS Foundation Trust; Royal Devon and Exeter Hospital; University Hospitals Coventry & Warwickshire; Yeovil District Hospital Foundation Trust; The Mid Yorshire Hospitals NHS Trust; Royal Hallamshire Hospital Sheffield; Aintree University Hospital; Eastbourne District General Hospital; Leeds Teaching Hospitals, NHS Trust; Queen Elizabeth the Queen Mother, East Kent; William Harvey Hospital; Aberdeen Royal Infirmary; Barking, Havering and Redbridge University Hospitals NHS Trust; University Hospital of North Durham; King's Mill Hospital; Luton & Dunstable NHSFT Hospital Trust; Morriston Hospital; Royal Devon & Exeter Hospital.

Vietnam: The People's Hospital 115; Viet Tiep Friendship Hospital; Bach Mai Hospital; Thanh Hoa General Hospital; Gia Dinh People's Hospital; University Medical Center, HCMC.

Supplementary Figure S1. Standardized mean differences plot for variables by the inverse probability of treatment weighting. An absolute standardized mean difference within an acceptable margin of 0.2 indicates well-balanced distributions of covariates. NIHSS denotes National Institute of Health Stroke Scale.

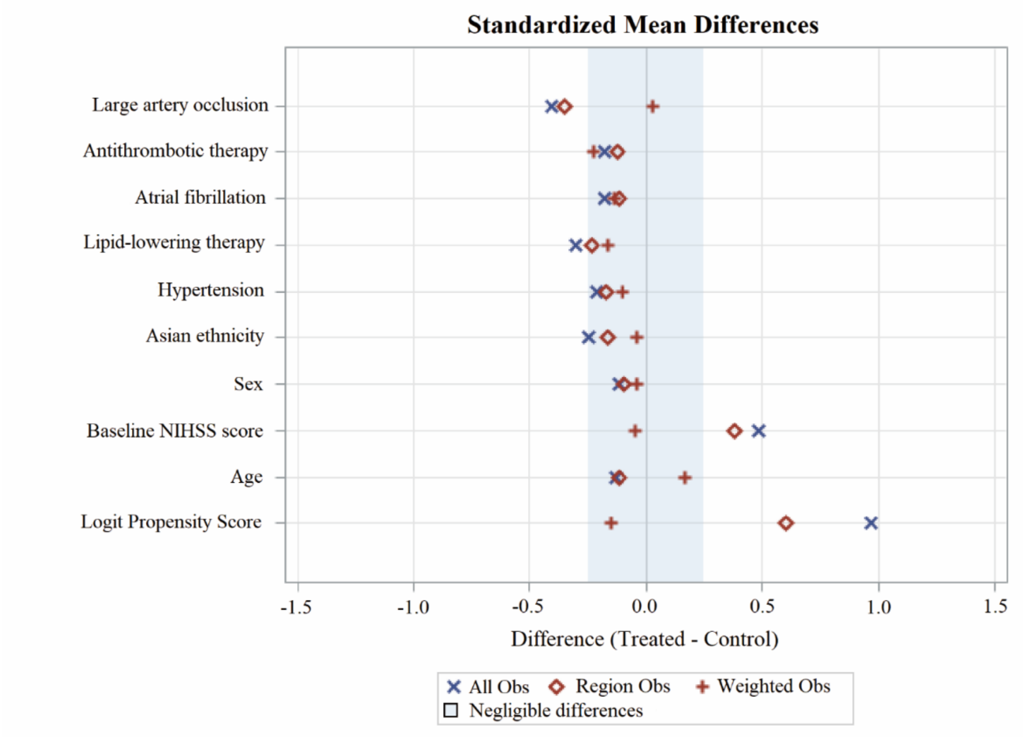

Supplementary Figure S2. Timing of decompressive hemicraniectomy. Of those surgical patients with available data of timing, there were 73 (99%) who underwent DHC within two days of neurological deterioration.

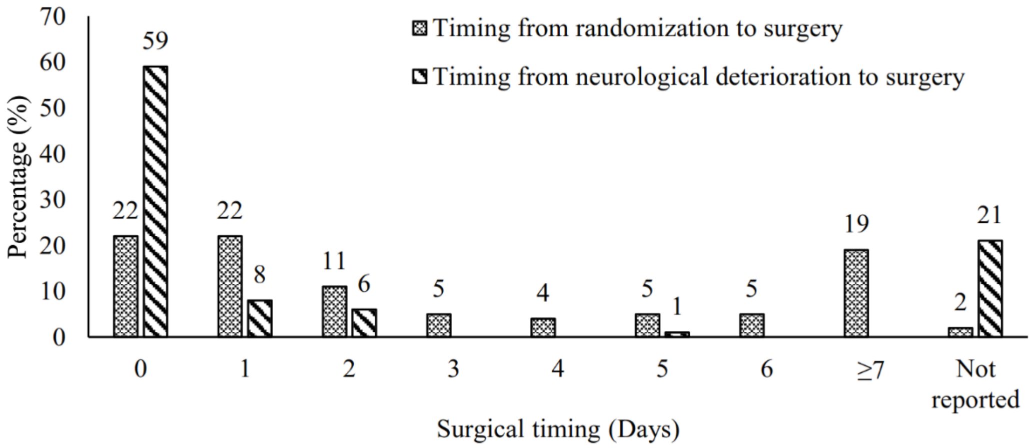

Supplementary Table S1. Clinical outcomes at 90 days in patients with different doses of intravenous thrombolytic treatment prior to decompressive hemicraniectomy.

	DHC with low-dose alteplase treatment (n=32)	DHC with standard-dose alteplase treatment (n=33)	OR (95% CI)	P value
Death/disability (mRS 2-6)*	28 (87.5)	25 (75.8)	0.45 (0.12-1.66)	0.23
Death/major disability (mRS 3-6)*	27 (84.4)	25 (75.8)	0.58 (0.17-2.01)	0.39
Death (mRS 6)*	11 (34.4)	11 (33.3)	0.96 (0.34-2.67)	0.93

CI confidence interval, DHC decompressive hemicraniectomy, mRS modified Rankin scale, OR odds ratio.

*mRS evaluates global disability; scores range from 0 (no symptoms) to 6 (death). A score of 2 to 5 indicates some degree of disability.

Supplementary Table S2. The ASPECTS in the ENCHANTED rtPA arm (Arm A) by DHC.

	DHC	
	Yes (n=62)	No (n=2854)
ASPECTS		
0-4	8 (12.9)	155 (5.4)
5-7	9 (14.5)	270 (9.5)
8-10	45 (72.6)	2429 (85.1)

ASPECTS Alberta Stroke Program Early CT Score, DHC decompressive hemicraniectomy.
